# Cytotoxic Th1 and Th17 cells infiltrate the intestinal mucosa of Behcet patients and exhibit high levels of TNF-α in early phases of the disease

**DOI:** 10.1097/MD.0000000000005516

**Published:** 2016-12-09

**Authors:** Giacomo Emmi, Elena Silvestri, Chiara Della Bella, Alessia Grassi, Marisa Benagiano, Fabio Cianchi, Danilo Squatrito, Luca Cantarini, Lorenzo Emmi, Carlo Selmi, Domenico Prisco, Mario Milco D’Elios

**Affiliations:** a Department of Experimental and Clinical Medicine, University of Florence; b Department of Surgery and Translational Medicine; c Research Center of Systemic Autoinflammatory Diseases and Behçet's Disease Clinic, Department of Medical Sciences, Surgery and Neurosciences, University of Siena; d SOD Interdisciplinary Internal Medicine, Center for Autoimmune Systemic Diseases –Behçet Center and Lupus Clinic – AOU Careggi; e Rheumatology and Clinical Immunology, Humanitas Research Hospital; f BIOMETRA Department, University of Milan, Milan, Italy.

**Keywords:** adalimumab, Behçet disease, biological, infliximab, secukinumab, Th cells, Th17

## Abstract

**Background:**

Gastrointestinal involvement is one of the most serious in Behçet disease, potentially leading to severe complications. Aim of this study was to investigate at mucosal level the T-cell responses in Behçet patients with early intestinal involvement.

**Methods:**

We isolated T cells from intestinal mucosa of 8 patients with intestinal symptoms started within 6 months. T lymphocytes were cloned and analyzed for surface phenotype and cytokines production.

**Results:**

We obtained 382 T-cell clones: 324 were CD4+ and 58 were CD8+. Within the 324 CD4+ clones, 195 were able to secrete IFN-γ and TNF-α, but not IL-4, nor IL-17 thus showing a polarized Th1 profile, whereas CD4 clones producing both IFN-γ and IL-17 (Th1/Th17 profile) were 79. Likewise, the number of CD8 clones producing type 1 cytokines was higher than those of CD8 clones producing both type 1 and 2 cytokines.

Almost all intestinal-derived T-cell clones expressed perforin-mediated cytotoxicity and Fas–Fas Ligand-mediated pro-apoptotic activity.

**Conclusions:**

Our results indicate that in the early stages of the disease, both Th1 and Th17 cells drive inflammation leading to mucosal damage via abnormal and long-lasting cytokines production as well as via both perforin- and Fas–Fas ligand-mediated cytotoxicity. Finally, all the T cells at mucosal level were able to produce large amount of TNF-α, suggesting that its production is a property of intestinal T cells of patients with early active intestinal disease. These results support the therapy with anti-TNF-α agents and suggest the use of anti-IL-17 monoclonal antibodies in Behçet patients with early intestinal involvement.

## 1 Introduction

Behçet disease (BD) is a systemic vasculitis characterized by mucocutaneous, ocular, and articular manifestations, as well as central nervous system, vascular, and gastrointestinal involvement. BD is common in the Middle East, Mediterranean countries, and Asia, while it is quite rare in the United States and Northern European countries.^[1]^ The exact cause of the disease remains unknown, but it is believed that genetic, immunological, and environmental factors contribute to its development.^[2]^ Recently, we have demonstrated that Behçet patients have a specific gut “microbiome signature.”^[3]^

T helper (Th) cells are considered the main effectors of adaptive immunity.^[4]^ T cells are able to differentiate into a Th1 profile in the presence of large amounts of interleukin (IL)-12 or into Th2 in the presence of high concentrations of IL-4.^[5]^ Recently other distinct classes of Th cells have been discovered, such as Th17 lymphocytes. Interestingly, Th17 and T regulatory (Treg) cells display the highest plasticity to switch to each other, and their balance seems to be of key importance to determine the progression or the cessation of autoimmune processes.^[4]^

T cells have a pathogenetic role also in BD; indeed recent works have shown an increased production of both Th1 and Th17 cytokines in peripheral blood, skin, and cerebrospinal fluid of Behçet patients.^[6–9]^ Moreover, it has been observed that anti-TNF-α treatment, and in particular the chimeric monoclonal antibody infliximab, is able to increase the proportion of Tregs, while inhibiting the differentiation of Th17 cells in Behçet patients with uveitis.^[10,11]^ Interestingly, Kim et al^[12]^ have recently observed that *IL-17A*, *IL-23R*, and *STAT4* polymorphisms could be involved in the pathogenesis of intestinal involvement in Behçet patients, almost in the Korean population.

To the best of our knowledge only 2 distinct papers have investigated the involvement of Th cells in the gut of Behçet patients, resulting in the evidence of a Th1-polarized response.^[13,14]^ Many patients with BD have intestinal symptoms (mainly diarrhea and abdominal pain), sometimes in the absence of specific colonoscopy findings; in the 5 nationwide surveys published in the past years on BD, gastrointestinal involvement ranges from 7.3% reported in Korean population to 16% described in Japan.^[15]^ In our country (Italy), the intestinal involvement is reported in about one third of patients, in accordance with our patients population.^[16]^ Notably, at the present time nothing is known about T cells infiltrating the intestinal mucosa of Behçet patients in the early stages of gut involvement.

On the basis of these considerations, we speculated that Th17 cells, together with Th1, are involved in the pathogenesis of the disease and investigated at mucosal level the type, role, and the cytotoxic potential of T-cell responses in BD with early gastrointestinal involvement, togather evidence for new biological treatments.

## 2 Material and methods

### 2.1 Patients enrollment and blood sample collection

From May 2012 to November 2014 8 consecutive patients with BD who attended the Florence Behçet Center (2 males and 6 females) and 10 healthy controls were included in the study. Patients with other autoimmune/autoinflammatory disorders, active infections, neoplasms, or Behçet patients on immunosuppressive treatment were excluded from the study. All the patients complained intestinal symptoms (abdominal pain and diarrhea) started within 6 months. The ileum bioptic specimens were collected from patients on treatment with prednisone  <10 mg/day and colchicine <2 mg/day. The biopsies were performed at the level of ulcers/aphtous lesions if present (3 patients), or at ileum level in absence of macroscopic lesions (5 patients). All the 8 patients with BD had evidence at histology of lymphomononuclear and neutrophilic inflammation at the site of ileal biopsies. None of these patients were under immunosuppressive or biological drugs at the time of biopsy. All the patients were diagnosed as having BD according to International Criteria for Behçet Disease.^[17]^ The study protocol was approved by local Ethical Committee, and informed consent was obtained from all subjects enrolled.

### 2.2 Generation of intestinal T-cell clones

Biopsy specimens were cultured for 7 days in RPMI 1640 medium supplemented with human IL-2 (50 U/mL; Eurocetus, Milan, Italy) to expand in vivo-activated T cells.^[18]^ Mucosal specimens were then disrupted, and single T-cell blasts were cloned under limiting dilution (0.3 cells/well), as described previously.^[19]^ The probability for each positive well being a clone derived from a single precursor was calculated by means of a conditional probability argument assuming Poisson statistics.^[20]^ The repertoire of the TCR Vβ chain of T-cell clones was analyzed with a panel of 22 mAbs specific to the following: Vβ1, Vβ2, Vβ4, Vβ7, Vβ9, Vβ11, Vβ14, Vβ16, Vβ18, Vβ20, Vβ21.3, Vβ22, and Vβ23 (Beckman Coulter); and Vβ3.1, Vβ5.1, Vβ5.2, Vβ5.3, Vβ6.7, Vβ8, Vβ12, Vβ13, and Vβ17 (AMS Biotechnology GmbH). Isotype-matched nonspecific Ig was used as negative control. Data acquisition was performed in a FACSCalibur™ flow cytometer using the CELLQuest™ software program (Becton Dickinson). From each T cell clone, mRNA was extracted by mRNA direct isolation kit (QIAGEN). For cDNA synthesis, the same amount of mRNA (50 ng) was used, and cDNA was synthesized by Moloney murine leukemia virus-reverse transcriptase (New England Biolabs, Inc.) and oligo-(dT) primers according to the manufacturer's protocol. cDNA mix of all samples was amplified under equal conditions by a 30-cycle PCR using Vβ T cell receptor typing amplimer kit (CLONTECH Laboratories, Inc.) according to the manufacturer's instructions. Evidence for clonality of the T cell clones was provided by the unique products of PCR analysis of TCR-Vβ mRNA expression obtained in clones or by the cytofluorimetric patterns of single TCR-Vβ expression shown by those clones. Each T cell clone was stained by only one of the TCR-Vβ chain-specific monoclonal antibodies, showing a single peak of fluorescence intensity. T-cell clones were analyzed by immunofluorescence on a fluorescence-activated cell-sorter after staining with the following: fluorescein isothiocyanate, phycoeritrin, or peridinin chlorophyll protein-conjugated mouse monoclonal antibodies (anti-CD4, anti-CD8, anti-CD69 purchased from Becton Dickinson, San Jose, CA). Virtually all T-cell clones showed individual patterns of response to superantigens (4 staphylococcal enterotoxins: SEA, SEB, SED, and SEE in the presence of allogeneic APCs), suggesting a difference in their T cell-reactive Vβ-chain expression.

### 2.3 Cytokine profile of intestinal T-cell clones

To assess the cytokines production, 10^6^ T-cell blasts of each clone were stimulated in duplicate cultures after stimulation for 36 hours with anti-CD3 monoclonal antibody or with PMA plus anti-CD3monoclonal antibody. Cell-free supernatants were collected and assayed for their cytokine content (IFN-γ, IL-4, IL-17, and tumor necrosis factor-α; Bio-Source International, Camarillo, CA) as previously described.^[21]^ Supernatants showing IFN-γ, IL-4, IL-17, and TNF-α levels 5 SD over the mean levels in control supernatants derived from irradiated feeder cells alone were regarded as positive. T-cell clones able to produce IFN-γ but not IL-4, nor IL-17 were categorized as Th1; clones able to produce IL-4 but not IFN-γ, nor IL-17 as Th2; clones producing IL-17, but not IFN-γ nor IL-4 as Th17; clones producing both IFN-γ and IL-17, but not IL-4 as Th17/Th1; and clones producing both IFN-γ and IL-4, but not IL-17 as Th0, as previously described.^[22]^

### 2.4 Perforin-mediated cytolytic activity

Perforin-mediated cytolytic activity of T-cell clones was assessed as previously reported.^[22]^ The ability of T-cell clones to express perforin-mediated cytotoxicity was investigated in a lectin-dependent assay against ^51^Cr-labeled P815 murine mastocytoma cells at effector-to-target ratios of 10, 5, and 2.5 to 1 in the presence of phytohemagglutinin (1% vol/vol), as previously described.^[22]^ After centrifugation to favor cell-to-cell contact, microplates were incubated for 8 hours at 37 °C, and 0.1 mL of supernatant was removed for the measurement of ^51^Cr release. Maximum release (MR) was obtained by treating target cells with 0.1 mL of 1 mol/L HCl. Spontaneous release (SR) was determined in microcultures without T cells. Specific lysis was calculated according to the formula: Percent Specific Lysis = 100 × (Experimental Release − SR)/(MR − SR). Cultures in which ^51^Cr release exceeded the mean SR by more than 5 SD were considered positive for cytolytic activity.

### 2.5 Fas–Fas ligand-mediated apoptotic killing

The ability of intestinal T-cell clones to induce Fas–Fas ligand-mediated apoptosis was assessed using Fas+ Jurkat cells as target.^[22]^ T-cell blasts from each clone were cocultured with ^51^Cr-labeled Jurkat cells at an effector-to-target ratio of 10, 5, and 2.5 to 1 for 18 hours in the presence of PMA (10 ng/mL) and ionomycin (1 mmol/L). Specific lysis was calculated according to the formula reported above.

### 2.6 Statistical analysis

Statistical comparison between groups was performed using the Mann–Whitney *U* test or the Wilcoxon test, as appropriate. Differences were considered as statistically significant when *P* < 0.05.

## 3 Results

### 3.1 Predominance of Th1 and Th1/Th17 lymphocytes in the intestinal mucosa

**Figure 1:**
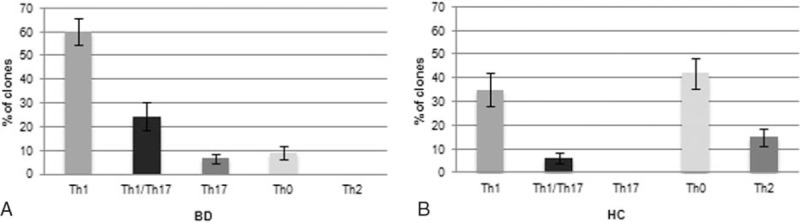
Cytokine profile of the CD4^+^ T helper clones derived from the intestinal mucosa of 8 patients with Behçet syndrome. Intestinal mucosa derived-T cell clones were obtained from 8 patients with BD and from 10 HCs. Duplicate samples of supernatants of mitogen-stimulated T cell clones were assayed for cytokine production. CD4^+^ clones able to produce IFN-γ, but not IL-4, nor IL-17 were categorized as Th1, CD4^+^ clones able to produce IL-17, but not IL-4, nor IFN-γ were categorized as Th17, CD4^+^ clones producing both IFN-γ and IL-17 were coded as Th1/Th17, CD4^+^ clones producing both IFN-γ and IL-4 were coded as Th0, and CD4^+^ clones able to produce IL-4, but not IFN-γ, nor IL-17 were categorized as Th2. BD = Behçet disease, CD = cluster of differentiation, HC = healthy control, IFN = interferon, IL = interleukin, Th = T helper.

**Figure 2:**
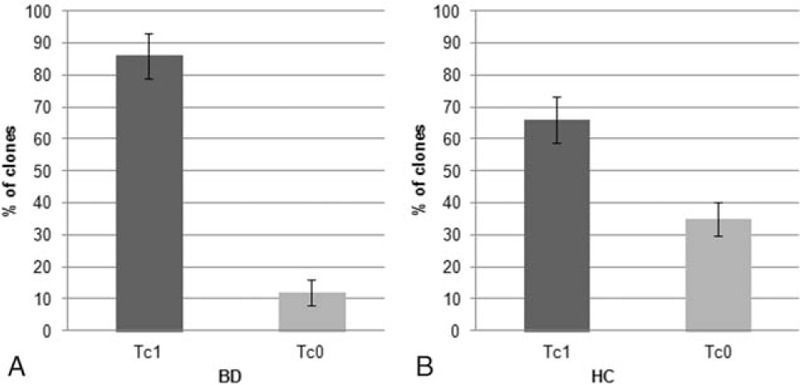
Cytokine profile of the CD8^+^ T cell clones derived from the intestinal mucosa of 8 patients with Behçet syndrome. Intestinal mucosa derived-T cell clones were obtained from 8 patients with BD and from 10 HCs. Duplicate samples of supernatants of mitogen-stimulated T cell clones were assayed for cytokine production. CD8^+^ clones able to produce IFN-γ, but not IL-4, were categorized as Tc1, whereas CD8^+^ clones producing both IFN-γ and IL-4 were coded as Tc0, respectively. BD = Behçet disease, HC = healthy control, IL = interleukin.

**Figure 3:**
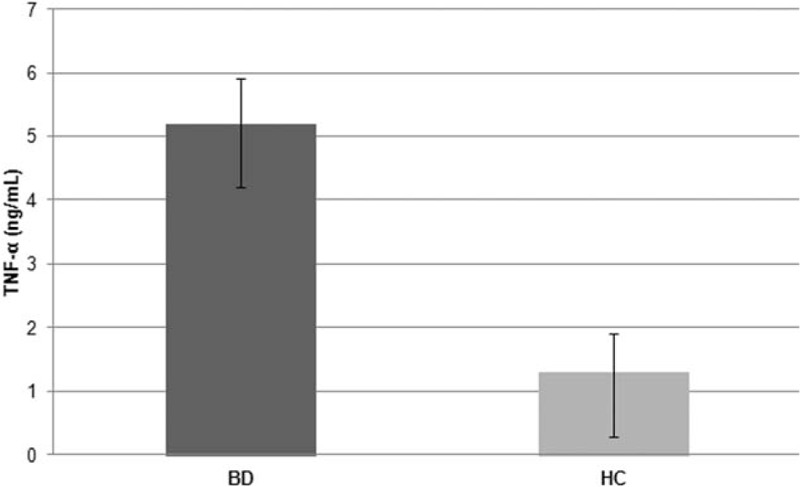
TNF-α production of the CD4^+^ T cell clones derived from the intestinal mucosa of patients with Behçet syndrome. Intestinal mucosa derived-T cell clones obtained from 8 patients with BD produced significantly (*P* < 0.05) less TNF-α than those obtained from the 10 HCs. Duplicate samples of supernatants of mitogen-stimulated T cell clones were assayed for TNF-α production. BD = Behçet disease, HC = healthy control, IL = interleukin.

T-cell clones (n = 382) were obtained from the intestinal mucosa of 8 patients with BD in order to investigate their phenotype in early phases of the disease: 324 were CD4+ and 58 were CD8+. All intestinal-derived clones were analyzed for their cytokine profile by measuring mitogen-induced production of IFN-γ, TNF-α, IL-4, and IL-17. In all BD patients, the majority (n = 195) of intestinal-derived clones were CD4 able to secrete IFN-γ and TNF-α but not IL-4, nor IL-17 thus showing a polarized Th1 profile (Fig. 1). CD4 clones producing IL-17, but not IFN-γ nor IL-4 were 21, whereas CD4 clones producing both IL-17 and IFN-γ (Th1/Th17 clones) were 79. CD4 clones able to secrete both IFN-γ and IL-4 (type 0 profile or Th0) accounted for only 29 clones. No clone from BD patients produced IL-4, but not IFN-γ nor IL-17 (Th2 clone). Likewise, in BD patients, the number of CD8 clones producing type 1 cytokines (Tc1 clones) (n = 50) was higher than those (n = 8) of CD8 clones producing both type 1 and 2 cytokines (Tc0 clones) (Fig. 2). From the intestinal mucosa of healthy controls (HC) we isolated 311 CD4+ clones and 42 CD8+ clones, respectively. Of note, all intestinal T-cell clones produced high concentrations of TNF-α (Fig. 3), particularly upon stimulation with PMA plus insoluble anti-CD3 antibody in the absence of APCs (mean ± SE, 5.2 ± 0.7 ng/mL per 10^6^ T cells), suggesting that high TNF-α production was a peculiar property of intestinal T cells of patients with BD, because under the same experimental conditions, in the series of intestinal T-cell clones from 10 healthy controls TNF-α-producing clones were 78%, with a mean (±SE) production of 1.3 ± 0.6 ng/mL per 10^6^ T cells (*P* < 0.05). It is of note that in healthy control the number of intestinal clones producing IFN-γ and/or IL-17 was significantly lower than in BD patients (*P* < 0.05) (Fig. 1).

### 3.2 Intestinal T-cell clones can induce death in target cells via both perforin- and Fas–Fas ligand-mediated pathways

**Figure 4:**
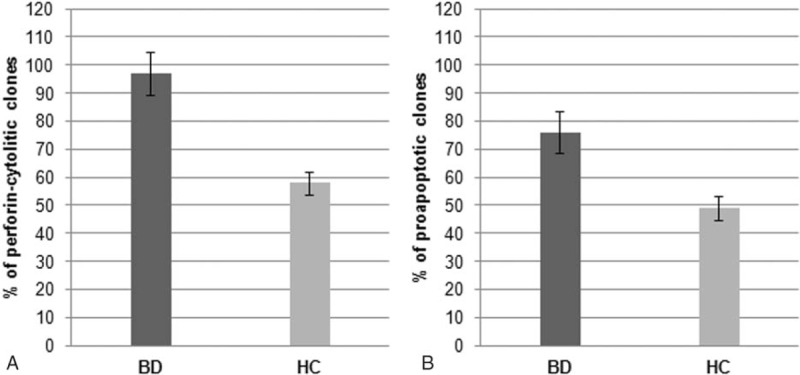
Cytotoxic and proapoptotic activity of intestinal mucosa-derived T cells from patients with Behçet syndrome. (A) Percentage of perforin-mediated cytotoxicity of Th clones derived from 8 patients with BD and from 10 HCs. To assess the perforin-mediated cytotoxicity, intestinal mucosa-derived T cell clones were cocultured at 10/1 effector-to-target ratios with^51^Cr-labeled P815 cells pulsed with PHA or medium alone, and ^51^Cr release was measured as index of specific target cell lysis. (B) Percentage of fas ligand-mediated apoptosis of Th clones derived from BD patients or HC. To assess the ability to induce apoptosis in target cells, intestinal mucosa-derived T cell clones were stimulated with mitogen or medium alone and were cocultured with ^51^Cr-labeled Fas^+^ Jurkat cells. The ^51^Cr release was measured as index of apoptotic target cell death. BD = Behçet disease, HC = healthy control, Th = T helper.

Because most activated Th1, Th17, and Th0 clones express perforin-mediated cytotoxicity^[23]^ the cytolytic potential of intestinal T-cell clones from patients with BD and HC was assessed by using PHA-pulsed ^51^Cr-labeled P815 as targets. At the effector-to-target ratio of 10 to 1, 315/324 (97%) Th clones of BD patients lysed target cells following PHA activation (Fig. 4A), whereas 58% Th clones of HC were indeed cytolytic. Because activated effector T cells can also kill their targets by inducing apoptosis through Fas–Fas ligand interaction,^[22]^ we evaluated the ability of activated intestinal clones to induce ^51^Cr release by Fas+ Jurkat cells undergoing apoptosis. On mitogen activation, 247/324 (76.2%) Th clones of BD patients were able to induce apoptosis in target cells (Fig. 4B) whereas 49% Th clones of HC were able to induce apoptosis. The role of Fas–Fas ligand interaction in this ^51^Cr release was confirmed by its inhibition (>50%) by a blocking anti-Fas antibody.

## 4 Discussion

In the present study, we clearly showed that in the early stages of active gut involvement of BD patients, cytotoxic Th1 and Th17 cells drive inflammation and that all the T cells at mucosal level are able to produce large amount of TNF-α, thus potentially leading to intestinal mucosal damage via different mechanisms.

Indeed, the intestinal manifestations of BD represent one of the most important involvement of the disease in terms of morbidity and mortality.^[24]^ Ileocecal ulcers are the most common among BD lesions, although any segment of intestinal tract could be damaged. Behçet patients may complain mild (abdominal pain, with or without diarrhea) to severe intestinal complications, mainly including perforation with hemorrhage and fistula formation.^[24]^ Systemic corticosteroids and traditional immunosuppressants have been used,^[25]^ but current evidence suggests that anti-TNFα biological agents are effective in BD^[26]^ and could be useful as standard therapy for moderate to severe gastrointestinal involvement of Behçet patients.^[26]^ Indeed, the use of human monoclonal antibody adalimumab has been recently approved in Japan for the treatment of BD patients with intestinal involvement.^[27]^ In the past, some studies have indirectly shown the production of TNF-α in patients with BD with intestinal involvement, but without any specific data on the duration of the disease.^[13,14]^ Of note, in our study we have shown that all the T cells infiltrating the intestinal mucosal of Behçet patients with early symptoms (abdominal pain and/or diarrhea started within 6 months), both with or without intestinal ulcers at endoscopy evaluation, are able to produce large amounts of TNF-α; this evidence supports the use of anti-TNF-α treatment, such as infliximab and adalimumab, also in the early stages of intestinal involvement, thus preventing the onset of more serious complications. Interestingly, it has been shown that anti-TNF-α treatments are able to exert their effects also increasing the proportion of Tregs, while inhibiting the differentiation of Th17 cells in Behçet patients with uveitis.^[10,11]^ This observation is very interesting in the light of our results; indeed anti-TNF-α could be effective in the early phases of gut involvement of Behçet patients due to both their direct capacity to inhibit the local production of TNF-α, as well as for their ability of modulate the T cells balance.

To date a Th1, but not a Th17 phenotype has been described in the gastrointestinal mucosa of BD patients.^[13,14]^ Indeed, in 2005 Imamura observed by immunohistochemistry analysis that cells infiltrating the intestinal lesions of Behçet patients were mainly constituted by CD4+T lymphocytes. Moreover, they detected in the intestinal lesions the upregulated expression of mRNA of IFN-α, Txk (a Th1-specific transcription factor) and CCR5 (a Th1-chemokine receptor), suggesting that a Th1 inflammation takes important part in BD patients. They also described in the intestinal mucosa of the same BD patients an increased expression of MIP1β, specific ligand for CCR5.^[13]^ In 2010 Ferrante et al^[14]^ have confirmed the relevance of Th1 response at the intestinal level of patients with BD. In particular, they observed at mucosal level the upregulation of mRNA for TNF-α, IFN-α, IL-12p35, and IL27; a significant increase of Th1-related cytokines was also detected in the peripheral blood of Behçet patients. More recently, *IL-17A*, *IL-23R*, and *STAT4* polymorphisms were described in BD patients with intestinal involvement, thus suggesting that also Th17 cells may participate to the mechanisms finally leading to the organ damage.^[12]^ In our study, we observed at mucosal level a great proportion of CD4+T cells; interestingly, these cells displayed a polarized Th1 or a Th1/Th17 phenotype. These results indicate for the first time that in early stages of intestinal involvement, both Th1 and Th17 cells drive inflammation in the intestinal mucosa of patients with BD and suggest that blocking their inflammatory-related cytokines might be useful for the treatment of the disease. Interestingly, targeting the Th17-related cytokines pathways has been proposed; a recent randomized control clinical trial conducted in 118 patients failed to demonstrate the superiority of the anti-IL17 secukinumab in the treatment of uveitis in Behçet patients.^[28]^ Nevertheless, blocking the IL17 axis could be important in the treatment of organ involvement other than ocular, and thus representing a novel promising treatment, at least for some disease phenotypes, such as the intestinal ones.

Moreover, we have shown for the first time that the T cells infiltrating the intestinal mucosa of Behçet patients are able to induce death in target cells via both perforin- and Fas–Fas Ligand-mediated pathways. Interestingly, a CD8^bright^CD56+ T cells population with cytolytic potential have been demonstrated in patients with active Behçet uveitis; these cells are able to exert their harmful effects through both Fas ligand-dependent and perforin-dependent pathways.^[29]^ Thus, also at the early stages of intestinal involvement, both CD4+ and CD8+ T cells are able to express their potential cytolytic damage in BD patients, via both perforin- and Fas–Fas ligand-mediated pathways.

The results obtained in this study indicate that in the early stages of BD gut involvement, both Th1 and Th17 cells drive inflammation. Moreover, those T cells, if inflammation proceeds unabated, may lead to mucosal damage via abnormal and long-lasting cytokine production as well as via both perforin- and Fas–Fas ligand-mediated cytotoxicity. Finally, all the T cells at mucosal level were able to produce large amount of TNF-α, suggesting that its production is a peculiar property of intestinal T cells of patients with early active intestinal disease. Overall the results obtained support the therapeutic approach with anti-TNF-α biological agents and suggest the possible use of anti-IL-17 monoclonal antibodies in BD patients with early intestinal involvement. This could allow to start an effective targeted therapy in the early stages of the disease, thus preventing the most severe and invalidating intestinal complications, such perforation with hemorrhage and fistula formation.
